# Mass spectrometry imaging of *Arabidopsis thaliana* with *in vivo* D_2_O labeling

**DOI:** 10.3389/fpls.2024.1379299

**Published:** 2024-05-31

**Authors:** Sumin Na, Young Jin Lee

**Affiliations:** Department of Chemistry, Iowa State University, Ames, IA, United States

**Keywords:** *Arabidopsis thaliana*, *in vivo* isotope labeling, mass spectrometry imaging, matrix-assisted laser desorption/ionization, arabidopsides, chloroplast lipids, epicuticular wax

## Abstract

The commonly used analytical tools for metabolomics cannot directly probe metabolic activities or distinguish metabolite differences between cells and suborgans in multicellular organisms. These issues can be addressed by *in-vivo* isotope labeling and mass spectrometry imaging (MSI), respectively, but the combination of the two, a newly emerging technology we call MSI*i*, has been rarely applied to plant systems. In this study, we explored MSI*i* of *Arabidopsis thaliana* with D_2_O labeling to study and visualize D-labeling in three classes of lipids: arabidopsides, chloroplast lipids, and epicuticular wax. Similar to other stress responses, D_2_O-induced stress increased arabidopsides in an hour, but it was relatively minor for matured plants and reverted to the normal level in a few hours. The D-labeling isotopologue patterns of arabidopsides matched with those of galactolipid precursors, supporting the currently accepted biosynthesis mechanism. Matrix-assisted laser desorption/ionization (MALDI)-MSI was used to visualize the spatiotemporal distribution of deuterated chloroplast lipids, pheophytin *a*, MGDGs, and DGDGs, after growing day-after-sowing (DAS) 28 plants in D_2_O condition for 3–12 days. There was a gradual change of deuteration amount along the leaf tissues and with a longer labeling time, which was attributed to slow respiration leading to low D_2_O concentration in the tissues. Finally, deuterium incorporation in epicuticular wax was visualized on the surfaces of the stem and flower. The conversion efficiency of newly synthesized C30 aldehyde to C29 ketone was very low in the lower stem but very high at the top of the stem near the flower or on the flower carpel. This study successfully demonstrated that MSI*i* can unveil spatiotemporal metabolic activities in various tissues of *A. thaliana*.

## 1 Introduction

Metabolomics is one of the key “omics” technologies to bridge the gap between phenotype and genotype (Matsuda et al., 2012). It has been used to investigate the metabolic responses of plants to biotic and abiotic stresses or annotate gene functions (Alseekh and Fernie, 2018). A popular tool of choice for metabolomics analysis is mass spectrometry (MS) with chromatographic separation, allowing for the detection and quantification of hundreds or thousands of chemical species present in biological systems. The current MS-based metabolomics analysis has two critical limitations. One is in typical sample preparation extracting metabolites from homogenized tissue samples in which the metabolite differences between different cells and suborgans are often ignored. The other is the fact that it provides only metabolite concentration information, not the actual metabolic activities. The former is addressed by mass spectrometry imaging (MSI) technique by micron-size direct sampling of metabolites from the tissue sections and visualizing metabolites at cellular resolution (Lee et al., 2010). The latter is addressed by introducing precursors with stable isotopes and tracing labeled metabolites (Jang et al., 2018). However, there has been very limited study of combining the two, MSI with *in-vivo* isotope labeling here referred to as MSI*i*, in plant systems.

In this study, we adopt deuterium oxide (D_2_O) labeling to explore the utility of MSI*i* in several tissues of *Arabidopsis thaliana*. Other stable isotope precursors previously utilized for MSI*i* include ^15^N-ammonium in maize root imaging (O’Neill and Lee, 2020) and [U-^13^C]glucose in phosphatidylcholine (PC) imaging in *Brassica* seeds (Romsdahl et al., 2021). Compared to other isotope labeling, D_2_O labeling has an advantage in plants as a global labeling agent because all hydrogens originate from water (Nett et al., 2018). All hydrogen atoms are fixed via photosynthesis and converted to nicotinamide adenine dinucleotide phosphate, a key biosynthetic intermediate from which all carbon-bound hydrogen atoms are derived. It has previously proven effective in the studies of protein turnover rate (Yang et al., 2010), tracing hormone metabolites (Åstot et al., 2000) in *A. thaliana*, and D-labeling of annual ryegrass (Evans et al., 2014) and switchgrass (Evans et al., 2015). D_2_O labeling, however, has not been used for MSI*i* other than our recent application to duckweed imaging (Tat and Lee, 2024) and cancer tissue imaging in mouse by the Northern group (Louie et al., 2013).

Because D_2_O labeling is commonly used in tracing fatty acid biosynthesis (Lee et al., 1994), lipids were the major metabolites of interest in our study, as they are also readily detected in matrix-assisted laser desorption/ionization (MALDI)-MSI. First, we investigated the effect of D_2_O on arabidopsides. The oxylipid arabidopsides are produced by the enzymatic oxidation of chloroplast galactolipids under a wide range of stress conditions (Vu et al., 2012; Genva et al., 2019). We have previously reported that arabidopsides are highly enriched in *feronia*, a mutant deficient in FERONIA, a receptor-like kinase in *A. thaliana* that functions broadly throughout plant development (Hansen et al., 2019b). We tried to test two hypotheses: one, whether D_2_O-induced stress increases arabidopsides as abiotic stress; two, whether the D-labeling isotopologue pattern matches that of galactolipid precursor. Second, D-labeled chloroplast lipids were visualized on leaves, specifically monogalactosyldiacylglycerol (MGDG), digalactosyldiacylglycerols (DGDGs), and chlorophyll *a*. Deuterium incorporation into these chloroplast lipids changed dramatically across the leaf development and D_2_O labeling time. Finally, D-labeled epicuticular wax, especially C29 ketone and C30 aldehyde, was visualized on the surface of the flower and stem. The conversion efficiency of the newly synthesized C30 aldehyde to C29 ketone provided insights into their biosynthesis rate throughout the plant.

## 2 Materials and methods

### 2.1 Hydroponic growing conditions

Hydroponic culture of *Arabidopsis* was performed by modifying the method of Van Delden et al. (2020). *Arabidopsis thaliana* (Col-0) wild-type seeds were washed in a 1-mL centrifuge tube with 20% Tween 20, 70% ethanol, and 100% ethanol in sequence. Each cycle was repeated three times, with each treatment lasting 5 min. Then, the seeds were transferred to 0.5× Hoagland medium (HM) in a 1-mL centrifuge tube and stored at 4°C in the dark for stratification. Three days later, the seeds were sown on agar-filled 200 μL PCR tubes that were precut at the bottom. Germination was allowed to occur by placing ~50 PCR agar holders on a 200-μL pipette tip holder in a 2-L beaker with 120 mL of 0.5× HM. The beaker was covered with transparent plastic wrap. The air vent was made by making small holes in the plastic wrap on day-after-sowing (DAS) 7–9, and the plastic wrap was removed on DAS 10. The plants were transferred to 15-mL centrifuge tubes on DAS 14 filled with 0.5× HM by placing the PCR tubes into the hole made in the centrifuge tube cap. Either a small plant growth tent (2 ft × 2 ft × 4 ft) or a plant growth chamber (AR-36L2; Percival, Perry, IA, USA) was used to grow the plants. For the tent, a dimmable 600-W LED grow light (VA600; ViparSpectra, Richmond, CA, USA) and a humidifier with a humidity controller were used to provide the light and humidity, respectively. For both conditions, the light intensity was ~160 μmol·cm^−2^·s^−1^, and the temperature and humidity were maintained at 21°C–24°C and 60%, respectively. The small plant growth tent was set up for a short-day condition (8 h light/16 h dark) for vegetative growth, and the plant growth chamber was set up for a long-day condition (16 h light/8 h dark) for flowering. The growth medium was replaced by a new medium once a week, and 0.5–1 mL of medium was added to 15-mL centrifuge tubes each day to supplement the water loss.

### 2.2 Sample preparation to measure arabidopsides

For the arabidopside experiment, the plants were transferred to new 15-mL centrifuge tubes filled with 35% D_2_O with 0.5× HM on DAS 28 and incubated for 30, 60, 180, and 540 min. The lipid extraction procedure utilized was based on a well-established method (Vu et al., 2012) with a minor modification. Up to eight leaves were harvested, cut into pieces, and quickly immersed in 3 mL 75°C isopropanol with 0.01% butylated hydroxytoluene (BHT) for 15 min. Then, 1.5 mL of chloroform and 0.6 mL of H_2_O were added and agitated for 1 h. The lipid extract was transferred to a new glass tube using a glass pipette. Four milliliters of chloroform:methanol (2:1, v/v) with 0.01% BHT was added to the sample, and the lipid extract was combined with the first extract after shaken for 30 min. This step was repeated three times and the final solution was stored at −80°C until direct infusion electrospray ionization (ESI)-MS analysis.

For the wounding experiments, the plants were grown until DAS 28, and the leaves were wounded by crimping with a tweezer three or four times across the midvein of the leaf (Hansen et al., 2019b) and harvested 15 min after the wounding. The lipid extracts were analyzed by the direct infusion ESI-MS method.

For the MS measurement of deuterated arabidopsides, the *fer* mutants were obtained from Hongqing Guo from the Department of Genetics, Development, and Cell Biology, Iowa State University. The plants were grown in the same way as the wild type in 0.5× HM until DAS 28 and incubated in 35% D_2_O medium for 12 days. The leaves were wounded as above and harvested for direct infusion ESI-MS for the lipid extract.

### 2.3 Sample preparation for MSI of chloroplast lipids in the leaves

For MALDI-MSI of chloroplast lipids, *A. thaliana* were transferred to new 15-mL centrifuge tubes filled with 35% D_2_O medium on DAS 28 and harvested after 3, 6, and 12 days. The fourth true leaf of each plant was selected and fractured to expose the middle layer of the leaf as described elsewhere (Klein et al., 2015). Briefly, the leaf was washed in H_2_O for 10 s, attached to a packing tape, dried in a vacuum for 2 h, enclosed the tape to attach both sides of the leaf to the tape, and passed through a rolling mill to make mechanical damage to the internal tissues. Then, the packing tape was pulled over to produce two separated half-leaves exposing the internal mesophyll layers. The top half layer (adaxial side) was attached to a microscope slide using a double-sided tape, followed by gold sputtering for 20 s at 40 mA (Cressington 108; Ted Pella, Redding, CA, USA) to provide conductivity to the surface and also as a MALDI matrix (Hansen et al., 2019a). Tissue samples were either analyzed immediately or stored at −80°C until the analysis.

### 2.4 Sample preparation for MSI of epicuticular wax on flower and stem

For MALDI-MSI of epicuticular wax, *A. thaliana* were grown in the plant growth chamber for a long-day condition and transferred to 15-mL centrifuge tubes filled with 35% D_2_O medium on DAS 14. After 3 days of labeling, the plants that had entered flower developmental stage C were selectively harvested. Stem samples were taken from three regions: bottom (near to root), middle, and top stem (near to flower). The flower and stem samples were attached to stainless steel target plates using conductive double-sided carbon tape (Nisshin EM, Tokyo, Japan). Forceps were used to attach the sample tissues onto the plate while minimizing contact with forceps to avoid physical damage. All samples were dried in a vacuum (~400 mTorr) for 75 min. An in-house ESI sprayer (Paulson et al., 2023) was attached to a TM sprayer nozzle (HTX Technologies, Chapel Hill, NC, USA) and used for spraying colloidal silver as a matrix after 4:1 dilution (v/v) with methanol. The distance was kept at 3 cm between the tip of the ESI sprayer and the sample plate. The following conditions were used for the automatic ESI spray: ESI voltage, +7 kV; sheath gas, 25 psi N_2_; matrix flow rate, 0.03 mL/min; and robotic arm movement, eight passes at 1,200 mm/min. Colloidal silver (99.99% pure silver, 0.65 nm; 20 ppm) was purchased from Purest Colloids, Inc. (Westampton, NJ, USA).

### 2.5 Mass spectrometry analysis and data processing

All mass spectrometry analysis was conducted using a Q-Exactive HF Orbitrap MS (Thermo Scientific, San Jose, CA, USA) with a MALDI/ESI dual source (Spectroglyph, Kennewick, WA, USA) equipped with a 349-nm laser (Explorer One; Spectra Physics, Milpitas, CA, USA). For the direct infusion ESI-MS analysis of arabidopsides, samples were diluted to 1:10 (v/v) using an ESI solvent of chloroform:methanol (3:2, v/v) with 0.1% acetic acid and analyzed in positive mode ESI at +3 kV. Ten microliters of the sample was injected through a loop injection at a flow rate of 10 μL·min^−1^ using the ESI solvent. Data were collected for the *m*/*z* range of 600–1,100 with a mass resolution of 120,000 at *m*/*z* 200. MS/MS analysis was performed for structural analysis under the same condition as direct infusion ESI-MS using extracts prepared as indicated above. The isolation window was 0.4 Da and collision energies were optimized for each metabolite. For the MALDI-MSI of chloroplast lipids and epicuticular wax, tissue samples were analyzed in positive mode with a mass resolution of 120,000 at *m*/*z* 200 and raster steps of 30–50 μm. Data were collected for the *m*/*z* range of 750–1,100 for chloroplast lipids and 300–600 for epicuticular wax, respectively.

Raw data were converted to imzML files using Image Insight (Spectroglyph) and loaded into the MSiReader (North Carolina State University; Raleigh, NC, USA) software (Robichaud et al., 2013). The average spectrum was obtained for the entire data using XCalibur (Thermo Scientific) or for the specific region of interest (ROI) using MSiReader and was used for the subsequent abundance or isotopologue analysis. For the visualization of the fractional abundance of deuterium, the *m*/*z* abundance and position data (X, Y) were exported into an Excel file using the MSiExport tool of MSiReader. This file was then imported into MATLAB (MathWorks, Natick, MA), and the fractional abundance of deuterium was visualized. ElemCor was used to deconvolute natural isotope contribution and obtain pure D-labeling isotopologue distributions (Du et al., 2019). A mass tolerance of 2 ppm was used to identify the monoisotope peaks, and MS images were produced with ±2.5 ppm, except for 3-day MS images, in which ±5 ppm was used due to highly abundant ^13^C isotopes.

## 3 Results

### 3.1 The effects of D_2_O on arabidopsides

**Figure 1:**
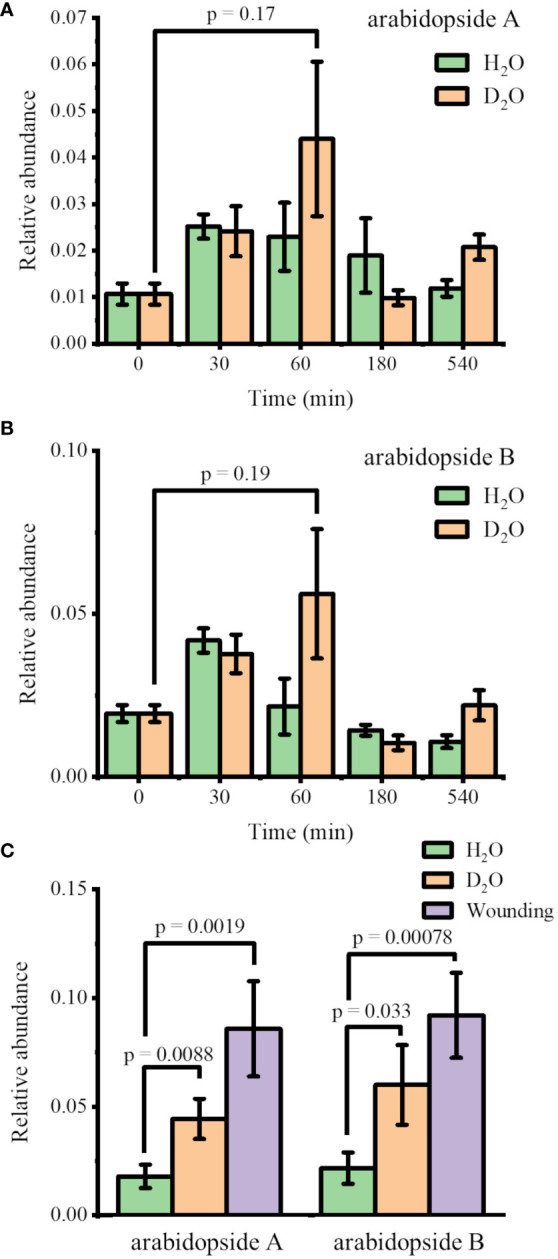
Change in the relative abundances of **(A)** arabidopside A and **(B)** arabidopside B in *Arabidopsis thaliana* after moving to H_2_O or 35% D_2_O medium (*n* = 3). **(C)** Comparison of the relative abundances of arabidopsides 1 h after moving to new media vs. 15 min after wounding (*n* = 7). All the abundances of arabidopsides A and B were normalized by their precursors, MGDG 34:6 and MGDG 36:6, respectively. Arabidopsides and MGDGs were all detected as Na^+^ adduct.

*Arabidopsis thaliana* plants were hydroponically grown in 0.5× HM until DAS 28 and transferred to new 0.5× HM with or without 35% D_2_O. The plants were harvested after four incubation times (30, 60, 180, and 540 min) to monitor the abundance changes in arabidopsides. The identity of these lipids was confirmed with MS/MS as shown in **Supplementary Figure S1** for arabidopside A, arabidopside B, and MGDG 34:6, matching with the literature (Hu et al., 2012; Hansen et al., 2019b). The direct infusion ESI-MS results are shown in **Figures 1A, B** for the relative abundance of arabidopside A and arabidopside B, respectively, the two most abundant arabidopsides known for significant increase upon wounding (Stelmach et al., 2001; Buseman et al., 2006; Vu et al., 2012; Hansen et al., 2019b). The abundance of arabidopsides A and B was normalized by their precursors, MGDG 34:6 and MGDG 36:6, respectively. Upon transferring to new media, arabidopsides A and B were slightly increased in both H_2_O and D_2_O media, peaking at 30 min and 60 min, respectively. While the change in the H_2_O medium was completely insignificant (*p* = 0.44 and 0.84 for arabidopsides A and B, respectively, at 60 min), the change in D_2_O showed a minute difference (*p* = 0.17 and 0.19 for arabidopsides A and B, respectively, at 60 min) compared to time 0. However, the difference was not significant, and the arabidopside abundance was reverted to initial levels in a few hours. This suggests that the observed changes may be partially attributed to a stress response from the transfer procedure, and the effect of D_2_O stress was rather minor to arabidopsides.

For further verification, we performed another experiment comparing the D_2_O stress response and the wounding response. **Figure 1C** shows the arabidopside abundance 15 min after wounding compared to 60 min after transferring to H_2_O or D_2_O medium. With the increase in sample size (*n* = 7), the abundance differences in arabidopsides A and B were now slightly significant (*p*< 0.01 and 0.05, respectively) when comparing 35% D_2_O and H_2_O. However, the abundance increase was much smaller than the increase of arabidopsides after wounding. We concluded that the D_2_O stress response was relatively minor compared to other abiotic stress such as wounding. A similar trend was observed for arabidopside D when comparing 60 min D_2_O incubation with time 0 or wounding response (**Supplementary Figure S2**). In contrast to arabidopsides A and B, however, arabidopside D had a higher abundance up to 180 min in both H_2_O and D_2_O. It should be noted that direct infusion ESI-MS is expected to be sufficient for the current purpose considering that the high mass resolution used in this study should be able to distinguish most interferences for these lipids, but further verification might be necessary with LC-MS to confirm the observed trend.

**Figure 2:**
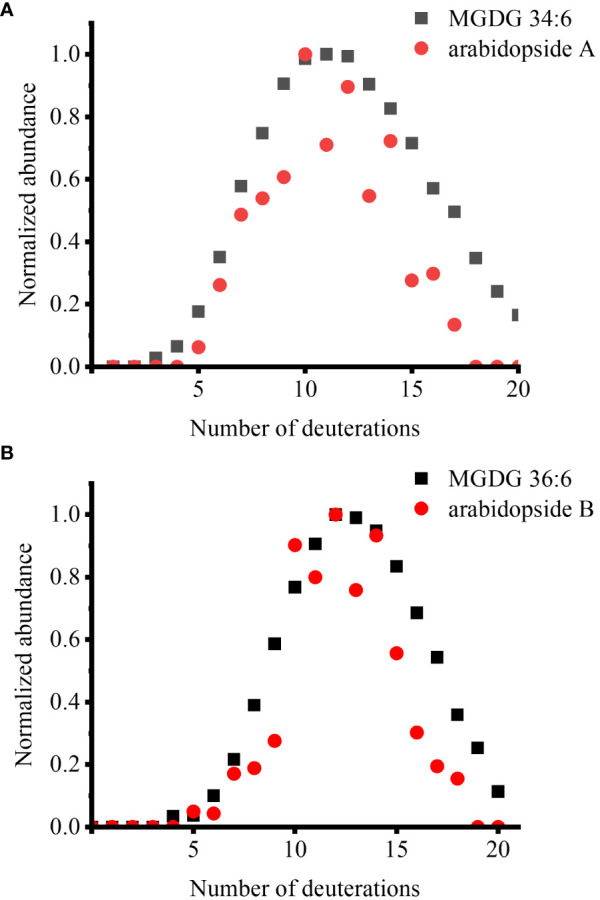
Comparison of deuterium incorporation in arabidopsides and their MGDG precursors in the *fer* mutant, which was incubated in 35% D_2_O medium for 12 days, after 15 min of wounding. **(A)** arabidopside A and MGDG 34:6 and **(B)** arabidopside B and MGDG 36:6. Arabidopsides were detected as Na^+^ adduct and MGDGs were detected as K^+^ adduct. ElemCor was used to deconvolute natural ^13^C isotopes.

We also sought to observe deuterated arabidopsides, but there were not enough signals detected for D-labeling within a few hours or even after a few days. This is attributed to the dilution of already low arabidopside signals into multiple isotopologues. After multiple trials, we could detect deuterated arabidopsides after wounding *fer* mutant with multiple days of labeling (**Figure 2**). We have previously reported that arabidopsides are highly enriched in *fer* mutant and increased further with wounding (Hansen et al., 2019b). After growing *fer* mutants in 0.5× HM until DAS 28, these mutants were incubated in 35% D_2_O medium for 12 days. The lipid extract from the leaves harvested after 15 min of wounding was subject to direct infusion ESI-MS analysis. When the isotopologue profiles were compared between the two arabidopsides and their MGDG precursors, they were very closely matched, showing a similar D-incorporation pattern (**Figure 2**). There was a slightly lower relative abundance for arabidopsides than that of precursors in high deuteration (e.g., D_15_ or higher), which is expected considering that arabidopsides have four fewer carbon-bound hydrogens than the precursors as can be seen in the binomial distribution simulation (**Supplementary Figure S3**).

### 3.2 Mass spectrometry imaging of D-labeled chloroplast lipids

Similar to the MSI*i* of duckweed with D_2_O labeling (Tat and Lee, 2024), we performed MSI*i* of *A. thaliana* with D_2_O labeling to visualize the chloroplast lipids on the leaves, specifically chlorophyll *a*, MGDGs, and DGDGs. The aim was to elucidate the spatial differences in their biosynthesis within the leaf tissues by monitoring deuterium incorporation into these lipids. *Arabidopsis thaliana* were grown in 0.5× HM until DAS 28, then transferred to 35% D_2_O medium for 3–12 days before being subjected to MALDI-MSI with the fracturing method (Klein et al., 2015). The fracturing method allows to split a leaf tissue into two halves across the longitudinal direction so that the internal mesophyll layers are exposed for interrogation by laser in MALDI-MSI. While tissue damage is unavoidable in this sample preparation, structural integrity was reported to have been mostly maintained at least at a resolution of ~10 µm in the SEM images. As shown in **Supplementary Figure S4**, a shift of mass spectral features was observed for the major lipids due to deuterium incorporation.

**Supplementary Figures S5D–F** show a series of MS images with various deuterium incorporation for MGDG 36:6, DGDG 36:6, and pheophytin *a* (chlorophyll *a* after losing Mg^2+^ during MALDI-MS) on the fourth true leaf of *A. thaliana* incubated in 35% D_2_O for 6 days. Interestingly, depending on the number of deuteration, there was a gradual change in localization from the tip of the leaf toward the base. In both galactolipids and pheophytin *a*, unlabeled monoisotope peaks (M0) were localized mostly at the tip of the leaf. As the number of deuteration increases, the distribution slowly propagates throughout the blades, with more or less even distribution for M6 or M7, then more localized toward the base for M12. MS images obtained after 12 days of D_2_O labeling showed similar patterns but with many more deuterations, with M10 or M11 being the most abundant (**Supplementary Figures S5G–I**). A similar behavior was observed for MS images obtained after 3 days of D_2_O labeling, although it was not as clear due to much less D-labeling and highly abundant unlabeled monoisotope (M0) and its ^13^C isotope (M1, M2) throughout the leaf (**Supplementary Figures S5A–C**). A similar trend was observed for other galactolipids, MGDG 34:6 and DGDG 34:6 (not shown).

**Figure 3:**
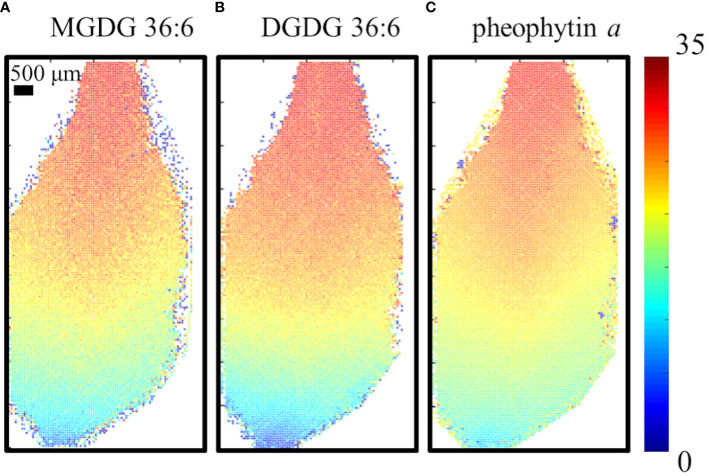
Visualization of the fractional abundance of deuterium, F_D-label_, for **(A)** MGDG 36:6, DGDG 36:6, **(B)** DGDG 36:6 and **(C)** pheophytin *a* on the fourth true leaf of *A. thaliana* incubated in 35% D_2_O for 6 days. All detected as K^+^ adduct.


To ensure the MS images of D-labeling are not artifacts due to the different levels of cell development in each cell, the fractional abundance of deuterium, F_D-label_, was calculated at each spot and visualized as shown in **Figure 3** for the 6-day D-labeling data. F_D-label_ can be calculated by the following equation and represents how much fraction of hydrogen is labeled out of the total hydrogens including those from the pre-existing unlabeled molecules (Larson et al., 2022).

$$F_{D-label}\;=\frac{({\text{MW}}_{{\text{D}}_{2}\text{O}}-{\text{MW}}_{{\text{H}}_{2}\text{O}})/\left(m_{D}-m_{H}\right)}{\left({\text{number of H}}_{\text{c}-\text{bound}}\right)\times \left({\text{D}}_{2}\text{O}\;\text{conc}.\right)}\times 100\;\left(\%\right)$$

where ${\text{MW}}_{{\text{D}}_{2}\text{O}}$ and ${\text{MW}}_{{\text{H}}_{2}\text{O}}$ represent the average molecular weights of the lipid species in D_2_O and H_2_O, respectively, and $m_{D}-\;m_{H}$ is the mass difference between a deuterium and a hydrogen atom, 1.00627 Da. The number of ${\text{H}}_{\text{c}-\text{bound}}$ refers to the number of hydrogen atoms bound to carbon within the lipid molecule that is available to be labeled by deuterium. Here, we considered only carbon-bound hydrogens because the washing step during the fracturing will provide the back exchange of exchangeable hydrogens (e.g., –OH). D_2_O conc. represents the concentration of D_2_O in the experiment, which is 35% in our experiment. The images of the F_D-label_ showed similar patterns for all three lipid species. F_D-label_ was close to 1.5% at the tip of the leaf but gradually increasing toward the base with ~32% at the very end of the base. This visualization removes the apparent cell-to-cell variation in raw signals, such as high abundance of galactolipids or low abundance of pheophytin *a* on the mid-vein (**Supplementary Figures S5D–F**). Almost no labeling at the leaf tip and the highest labeling at the leaf base coincide with the fact that the leaf base is the cell proliferation zone with active cell growth while the leaf tip is the matured zone with almost no new cells.

As D-labeling was most active at the leaf base, we calculated the D-labeling efficiency of five major lipids for 3, 6, and 12 days of D_2_O labeling with the base of the leaf as the region of interest (ROI), as indicated in **Supplementary Figure S6**. It is similar to F_D-label_ but excluding pre-existing unlabeled molecules and can be calculated using the following equation (Larson et al., 2022):


$$\text{D}-\text{Labeling efficiency}\;=\frac{\text{Average number of D}}{\left({\text{number of H}}_{\text{c}-\text{bound}}\right)\times \left({\text{D}}_{2}\text{O}\;\text{conc}.\right)}\times 100\;\left(\%\right)$$

**Figure 4:**
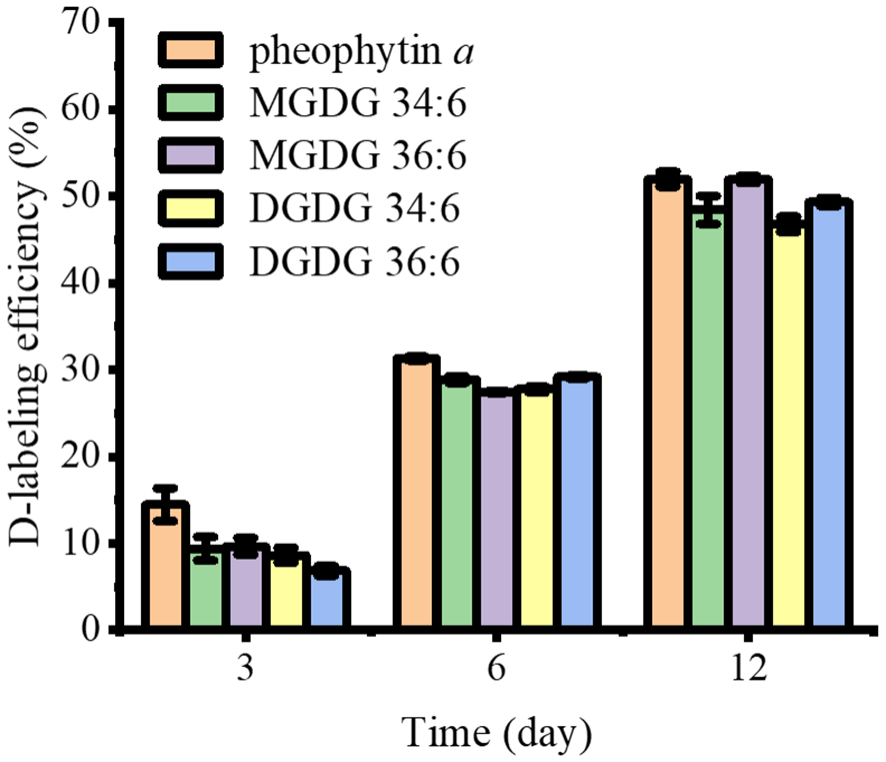
The comparison of D-labeling efficiency of pheophytin *a*, MGDGs, and DGDGs in the leaf base after 3–12 days of D_2_O labeling (*n* = 3). All detected as K^+^ adduct. Contribution from the natural ^13^C isotope was deconvoluted using ElemCor.

D-labeling efficiency is calculated using a fraction showing the average number of deuterium that can be labeled compared to the quantity that is theoretically possible. One technical consideration is the fact that there are significant ^13^C_1_- and ^13^C_2_-natural isotope contributions that cannot be separated from D_1_- and D_2_-labeling with the mass resolution used in this study. The ElemCor software (Du et al., 2019) was used to deconvolute this natural isotope abundance and obtain pure D-labeling efficiencies. As shown in **Figure 4**, pheophytin *a* had a D-labeling efficiency of 14% on day 3, which increased to 31% on day 6 and increased further to 52% on day 12. In contrast, D-labeling efficiency was much lower than that of pheophytin *a* for all galactolipids on day 3, 7%–10%, but it increased to a similar level with pheophytin *a* by day 6 and after.

### 3.3 D-labeling on epicuticular wax in the flower development

**Figure 5:**
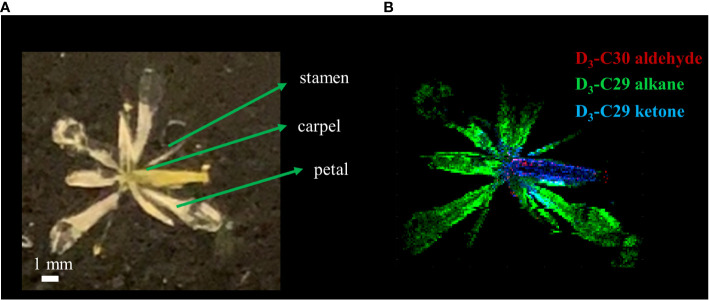
**(A)** Optical and **(B)** MALDI-MS images of *Arabidopsis thaliana* flower after 3 days of D_2_O labeling on DAS 14. MS images were obtained on the surface of the flower as silver ion adducts, [M+^107^Ag]^+^.

**Figure 6:**
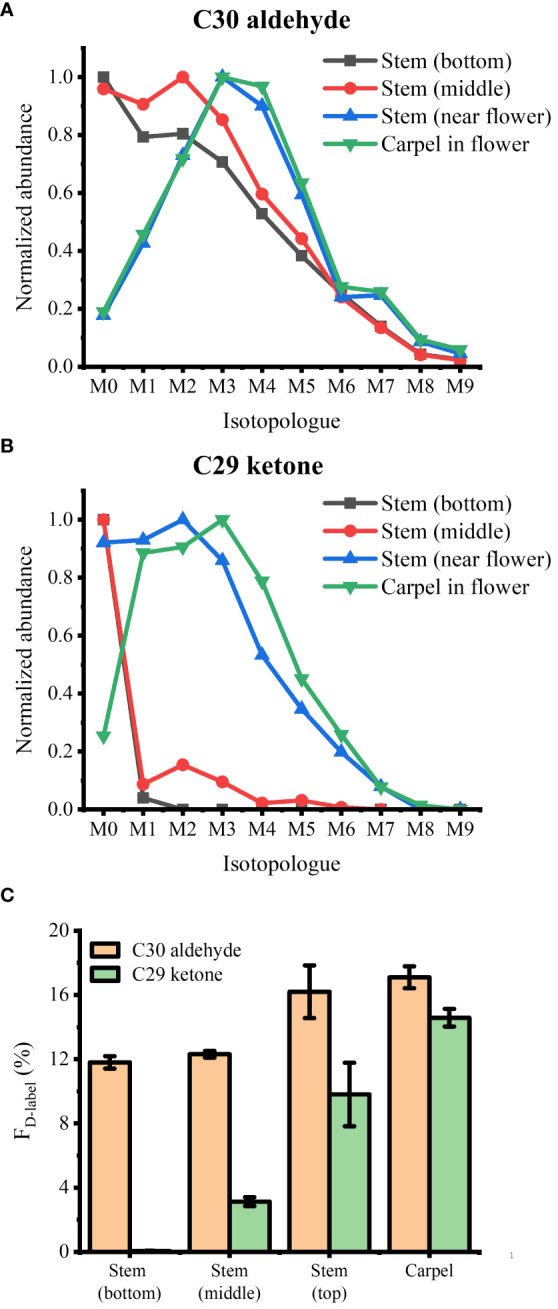
Isotopologue distributions of deuterated **(A)** C30 aldehyde and **(B)** C29 ketone and **(C)** their fractional abundance of deuterium, F_D-label_, in various parts of *Arabidopsis thaliana* after 3 days of D_2_O labeling (*n* = 3). All detected as ^107^Ag^+^ adduct. Contribution from the natural ^13^C isotope was deconvoluted using ElemCor.

As a last example, we applied D_2_O labeling to the epicuticular wax on the flower and various parts of the stem. In the long-day condition, *A. thaliana* were transferred to 35% D_2_O medium on DAS 14 approximately 3 days before flowering. After 3 days of labeling, the plants were harvested that had entered flower developmental stage C, where emerging petals are perpendicular to the flower axis, resulting in a clear physical separation from the adjacent tissues. As we have demonstrated previously (Jun et al., 2010), the use of colloidal silver as a matrix can ionize hydrophobic epicuticular wax as silver ion adducts and visualize their localization across the flower surface with MALDI-MSI. The mass spectra of D-labeled C29 alkane and C29 ketone are shown in **Supplementary Figure S7**. **Figure 5** shows the MS images of D_3_-labeled C30 aldehyde, C29 alkane, and C29 ketone on *A. thaliana* flower. Successful deuterium incorporation in just 3 days of labeling indicates that these surface lipids are synthesized rapidly during the flower developmental stage. D-labeled metabolites on each tissue of the flower showed unique distribution. C29 alkane was the most abundant on the petal and stamen and widely distributed among the tissues. In contrast, C29 ketone and C30 aldehyde were localized on the carpel of the flower. It is consistent with the previous report except for C30 aldehyde, which was not detected in the previous work due to the low mass resolution (Jun et al., 2010). In a similar experiment for **Figure 6**, various parts of the stem (bottom, middle, near the flower) as well as the flower were harvested to interrogate with MALDI-MSI. **Supplementary Figure S8** shows the MS images of C29 ketone with various amounts of deuteration on the flower and the middle section of the stem. In both the flower and mid-stem, up to six or seven deuterations could be observed, but three D-labeling (M3) was the most abundant in the flower, but unlabeled C29 ketone (M0) was the most abundant in the mid-stem, which is not surprising considering that there must be a significant amount of pre-existing epicuticular wax in the stem before being transferred to the D_2_O medium. **Figures 6A, B** show the isotopologue patterns of C29 ketone and C30 aldehyde (a precursor of C29 ketone) on various parts of the stem and the carpel of the flower. Overall, there was a high level of deuterium incorporation into C30 aldehyde in most tissues, but there was no or very little deuterium incorporation into C29 ketone in the mid or bottom part of the stem. This dramatic change between the lower parts of the stem vs. near or on the flower can be more quantitatively compared using the F_D-label_, shown in **Figure 6C**. F_D-label_ for C30 aldehyde was already ~12% in the bottom and mid stem after 3 days of D_2_O labeling, slightly lower than the top part of the stem and the flower, 16%–17%. However, there was only 0%–3% of F_D-label_ for C29 ketone in the lower stem, but ~10% and ~15% on the top part of the stem and flower, respectively. In other words, the conversion ratio of C30 aldehyde to C29 ketone was ~20% or less on the lower stem but 60% to 90% on the top part of the stem and flower.

## 4 Discussion

### 4.1 Hydroponic culture with 35% D_2_O provides significant but minor stress to *Arabidopsis*

For the first time, D_2_O labeling was successfully applied to the MSI*i* of *A. thaliana*, a terrestrial plant, using a hydroponic culture. Although unnatural for terrestrial plants, hydroponic culture is commonly used for D_2_O labeling of *A. thaliana* to precisely control isotope concentrations (Åstot et al., 2000; Yang et al., 2010). Van Delden and coworkers performed a systematic investigation on the effect of nutrient solutions in the hydroponic culture of *A. thaliana* (Van Delden et al., 2020). Nutrients with too high salt concentrations, such as in Murashige and Skoog, resulted in low biomass on DAS 48. Among the best performing nutrients they reported, we adopted 0.5× HM for hydroponic culture in this study. High D_2_O concentration is toxic to any biological organisms and gradually inhibits the root development of *Arabidopsis* as the D_2_O concentration increases from 0% to 40% (Yang et al., 2010). A concentration of 30% D_2_O significantly altered the gene expression in the short term (4 h) compared to the long term (7 days), indicating an adaptation to D_2_O-induced stress (Evans and Shah, 2015). To avoid the adverse effect in root development by D_2_O-induced stress, *A. thaliana* was grown to DAS 14 or 28 in hydroponic culture before transferring to 35% D_2_O medium in this study.

Before we performed MSI*i*, we first studied the effect of D_2_O on arabidopsides. A D_2_O concentration of 35% was used in all the experiments to maximize D-labeling, but it may induce abiotic stress. Mostly known as a wounding response, previous studies have reported that various stresses resulted in the accumulation of arabidopsides in *A. thaliana* in less than 1 h (Stelmach et al., 2001; Buseman et al., 2006; Vu et al., 2012). Another study reported that *A. thaliana* in 30% D_2_O altered gene expression related to wounding, with 16 genes upregulated and one gene downregulated after 4 h of growth (Yang et al., 2010). It is not previously known, however, whether D_2_O would increase arabidopsides as abiotic stress. Considering previous reports, we hypothesized that D_2_O-induced stress response may result in an increase of arabidopsides. Albeit slight, arabidopsides A and B were increased initially, supporting our hypothesis, but reverted to the normal level within a few hours (**Figure 1**). The maximum increase after 60 min in the D_2_O medium was twice less than the increase induced by the wounding response, suggesting that D_2_O-induced stress might be relatively minor and may not have serious long-term consequences. In fact, there was no apparent difference between non-labeled vs. labeled plants even after 12 days of labeling.

We tried all our efforts to visualize deuterated arabidopsides, but unfortunately, the amount of arabidopsides was so low that they were not detected by MALDI-MSI. It is a downside of MSI*i* with D_2_O labeling because D-labeled metabolites can often be detected only for major species because the binomial distribution of H- vs. D-labeling results in the dilution of D-labeled metabolites to a wide isotopologue distribution with multiple deuterations. Deuterated arabidopsides could be finally detected by combining multiple strategies without visualization, including 1) direct infusion ESI-MS, 2) 12 days of D_2_O labeling, 3) use of *fer* mutant, 4) wounding, and 5) combining multiple leaves. When deuterium isotopologue distributions were compared, deuteration patterns were very closely matching between arabidopsides and their precursors (i.e., MGDG 34:6 vs. arabidopside A, MGDG 36:6 vs. arabidopside B) (**Figure 2**). These data support a previous report that lipoxygenase oxidizes both fatty acid chains in MGDGs to form arabidopsides after wounding (Stelmach et al., 2001).

### 4.2 D-labeling of chloroplast lipids shows gradual deuteration from the leaf tip to the base

In the second set of experiments, D-labeled chloroplast lipids were successfully visualized in MSI*i* with D_2_O labeling for 3, 6, and 12 days. To our surprise, the MS images of MGDG, DGDG, and pheophytin *a* showed gradual changes across the leaf tissues depending on the fractional abundance of deuterium (**Figure 3**) or the number of deuterations (**Supplementary Figure S5**). Furthermore, their D-labeling efficiencies at the leaf base increased slowly from day 3 to days 6 and 12 of D_2_O labeling (**Figure 4**). To explain the gradual spatiotemporal change in D-labeling of the chloroplast lipids, we hypothesize that 1) the internal D_2_O concentration changes very slowly over many days and 2) there is a D_2_O concentration gradient across the entire plant. Water is a precious resource to terrestrial plants, and it is released mostly through the stomata with a tight regulation. Epicuticular wax covers all the air-exposed plant surfaces, protecting water evaporation in other places. The transpiration rate seemed to be very low in the given condition because we had to supplement only 0.5–1.0 mL of medium per day. As a result, its internal D_2_O concentration would not change immediately when the plants were transferred to 35% D_2_O medium but increased slowly over many days with a gradient across the entire plant. Accordingly, the amount of D-labeling in the chloroplast lipids would be subject to available D_2_O concentration at a given cell at the time of their synthesis. The low D-labeling at the leaf tip is attributed to 1) the lower D_2_O concentration than that at the leaf base and 2) being mostly “old tissues” synthesized when the D_2_O concentration was even lower. Another explanation is a higher flux of these lipids at the leaf base than at the leaf tip, which is supported by the fact that the leaf base is a proliferation zone with active cell growth. While it can explain the gradual change of D-labeling across the leaf tissues, it cannot explain the increase of D-labeling efficiency over time, suggesting that the D_2_O concentration gradient might be the main reason for the spatiotemporal change in D-labeling.

The gradual change of D-labeling across the leaf tissues is in contrast to our recent work in D_2_O labeling of duckweeds (*Lamna minor*) (Tat and Lee, 2024), in which three distinct isotopologue groups of galactolipids were found for the first few days of labeling due to the partial D-labeling of structural moieties. Their MS images, however, were essentially identical for the same isotopologue groups, localized to parent frond tissues for galactose-only D-labeling, intermediate tissues for galactose and a fatty acyl chain D-labeling, and newly grown daughter frond tissues for the D-labeling of the entire molecule. It is because *L. minor*, as an aqua plant, has its fronds fully in contact with water on the abaxial side and, thus, has the same D_2_O concentration across its fronds. Unlike the D_2_O labeling of duckweeds, we could not observe the separation of each isotopologue group in the D_2_O labeling of *Arabidopsis*, which is attributed to the combination of low signals, a lower D_2_O concentration (35% vs. 50%), and a lower D-labeling efficiency (~50% vs. ~97%).

Another interesting observation is that pheophytin *a* had a higher D-labeling efficiency than galactolipids on day 3, but similar on days 6 and 12 (**Figure 4**), although not significant (*p* = 0.08~0.13) except for DGDG 36:6 (*p* = 0.04) due to the low sampling size (*n* = 3). In our previous D_2_O labeling experiments of duckweeds (Tat and Lee, 2024), pheophytin *a* showed only one isotopologue pattern corresponding to the D-labeling of the entire molecule even in the very early days of labeling unlike galactolipids, which was attributed to the fast biosynthesis of pheophytin *a*. Similar to duckweed, we expect that pheophytin *a* would be fully labeled by day 3 in *A. thaliana* due to its fast biosynthesis, only limited by the low cellular D_2_O concentration, but newly synthesized galactolipids might be a mixture of partial and entire molecule labeling on day 3, although there is no clear separation among isotopologue groups, resulting in a low apparent D-labeling efficiency when averaged together.

### 4.3 D-labeling of epicuticular wax shows tissue-specific metabolic conversion difference

As a last example, D-labeled epicuticular wax was imaged on the stage C flower and several different locations of the stems (**Figure 5**, **Supplementary Figure S8**). These lipids with very long-chain fatty acids (VLCFAs) have a crucial role in forming the barrier on the outer plant surface (Yeats and Rose, 2013) and change dynamically during the flower’s developmental stages (Alexander et al., 2021). The three particular lipids that are visualized, C30 aldehyde, C29 alkane, and C29 ketone, are in the same alkane-forming pathway (Jenks et al., 2002). While C29 alkane was the most abundant among all surface lipids in *A. thaliana* and could be detected as a silver ion adduct in MALDI-MS using colloidal silver as a matrix, the ionization efficiency was very low and deuterated C29 alkane could be detected only in the flower but not in the stems.

When the relative abundances of deuteration were compared between C29 ketone (final product) and C30 aldehyde (a precursor of C29 ketone), the conversion ratio of newly synthesized C30 aldehyde to C29 ketone was the highest on the carpel followed by the top part of the stem near the flower, ~90% and ~60%, respectively, but very low on the middle and low parts of the stem, as determined by the fractional abundance of deuterium (**Figure 6**). The highest conversion rate on the carpel suggests the important role of C29 ketone in the reproduction of *A. thaliana*. It is intriguing why the conversion rate is very high on the top part of the stem, while very low in the middle or bottom part of the stem, which is in contrast to the lignin biosynthesis on *Arabidopsis* stems. Wang and coworkers reported that the incorporation of ^13^C_6_-Phe was most active near the base of the stem than in the top when cut stems were incubated with the medium supplemented by ^13^C_6_-Phe (Wang et al., 2018). Our result suggests that the enzymes involved in the conversion of C30 aldehyde to C29 ketone (aldehyde decarbonylase, alkane hydrolase, or alcohol oxidase) may not be strictly tissue type-specific but rather have high expression near the flowers.

### 4.4 Broad implication and limitation of this study

MSI*i* can elucidate the fine details of tissue-specific or cell-specific metabolism more than MSI or isotope tracing alone can offer. For example, by monitoring M3 vs. M6 UDP-glucose as a marker for glycolysis vs. gluconeogenesis, differential metabolic activity could be observed between the cortex and medulla in MSI of mouse kidney by infusing [U-^13^C]glycerol or [U-^13^C]glucose (Wang et al., 2022). In plants, there have been limited MSI*i* studies reported so far using stable isotopes. The MSI of developing seeds of camelina and pennycress labeled with [U-^13^C]glucose showed a higher ^13^C-labeling in the cotyledons compared to the embryonic axis (Romsdahl et al., 2021). They also observed a higher isotope enrichment in PC species with more saturated and longer chain fatty acids, which was attributed to more rapid fatty acid elongation than desaturation. Using D_4_- and ^13^C_9_-Tyr, new metabolites involved in Tyr metabolism were discovered and visualized in *Spirodela polyrhiza* (Feldberg et al., 2018). Genotypic and developmental differences in free amino acids were visualized in MSI of maize root cross-sections (O’Neill and Lee, 2020), in which ^15^N-ammonium was used to differentiate between external (^15^N from media) and internal (^14^N from seeds) nitrogens. Nitrogen-containing specialized metabolites were visualized in *Catharanthus* using ^15^N-labeling (Nakabayashi et al., 2017). As discussed in the prior section, 50% D_2_O labeling of duckweeds showed partial labeling of galactolipids and revealed their spatiotemporal changes (Tat and Lee, 2024). Many more MSI*i* studies are expected in the near future to unveil plant metabolic biology in unprecedented spatiotemporal details.

The current MSI*i* study of *Arabidopsis* confirms some of the strengths and weaknesses of this technological platform, specifically with D_2_O labeling. A low sensitivity is a critical obstacle in MSI in general hampered by micron-size small sampling size in each pixel, which is exacerbated in MSI*i* because the same metabolite is split among isotopologues. It is particularly worse in D-labeling compared to ^13^C or ^15^N because the maximum D_2_O concentration is limited to 35%–50% due to toxicity, resulting in a wide isotopologue distribution with various degrees of partial labeling. D_2_O-induced stress is another limitation in D-labeling, as it may lead to a potential artifact. It is virtually non-existent in ^13^C- or ^15^N-labeling, with the minimum kinetic isotope effect for heavy isotopes. The most benefit of D-labeling in MSI*i*, especially in plants, is that D_2_O is the sole source of all hydrogens in plants and is easy to incorporate in hydroponic culture. It is in contrast to ^13^C or ^15^N. A completely sealed growth chamber is required for long-term ^13^CO_2_ labeling while [U-^13^C]glucose enters carbon metabolism almost exclusively through glycolysis. ^15^N-labeling should take into account a complication coming from nitrogen fixation or transportation difference between ammonium and nitrate and among plant species.

MSI or MSI*i* of primary metabolites is very difficult due to their low ionization efficiencies and many possible structural isomers. Instead, lipids are most commonly interrogated by MSI including this work, thanks to their high abundance in cell membranes, minimum loss and less diffusion during the sample preparation, and a much smaller number of structural isomers. D_2_O labeling is particularly attractive for the isotope tracing of lipids as successfully demonstrated for *Arabidopsis* in this work and previously for duckweeds. While many isomers are still possible for the lipid species with the same molecular formulae depending on fatty acid chain length, sn-position, and double-bond position, many technical advancements are being made to resolve this issue including MS/MS imaging (Sun et al., 2023), MSI with ion mobility separation (Jiang et al., 2023), and ozone (Claes et al., 2021) or other chemical reactions (Li et al., 2024) to determine the double-bond position.

## Data availability statement

The raw data supporting the conclusions of this article will be made available by the authors, without undue reservation.

## Author contributions

SN: Data curation, Formal Analysis, Investigation, Methodology, Writing – original draft, Writing – review & editing. YL: Conceptualization, Funding acquisition, Methodology, Project administration, Resources, Supervision, Writing – original draft, Writing – review & editing.
