# Supplementary material for: Mass spectrometry imaging of Arabidopsis thaliana with in vivo D2O labeling
Source: Front Plant Sci. 2024 May 31;15:1379299. doi: 10.3389/fpls.2024.1379299 (PMC11176549; doi:10.3389/fpls.2024.1379299)
Supplement: Supplementary file 1 [file DataSheet_1.pdf]

Supplementary Material

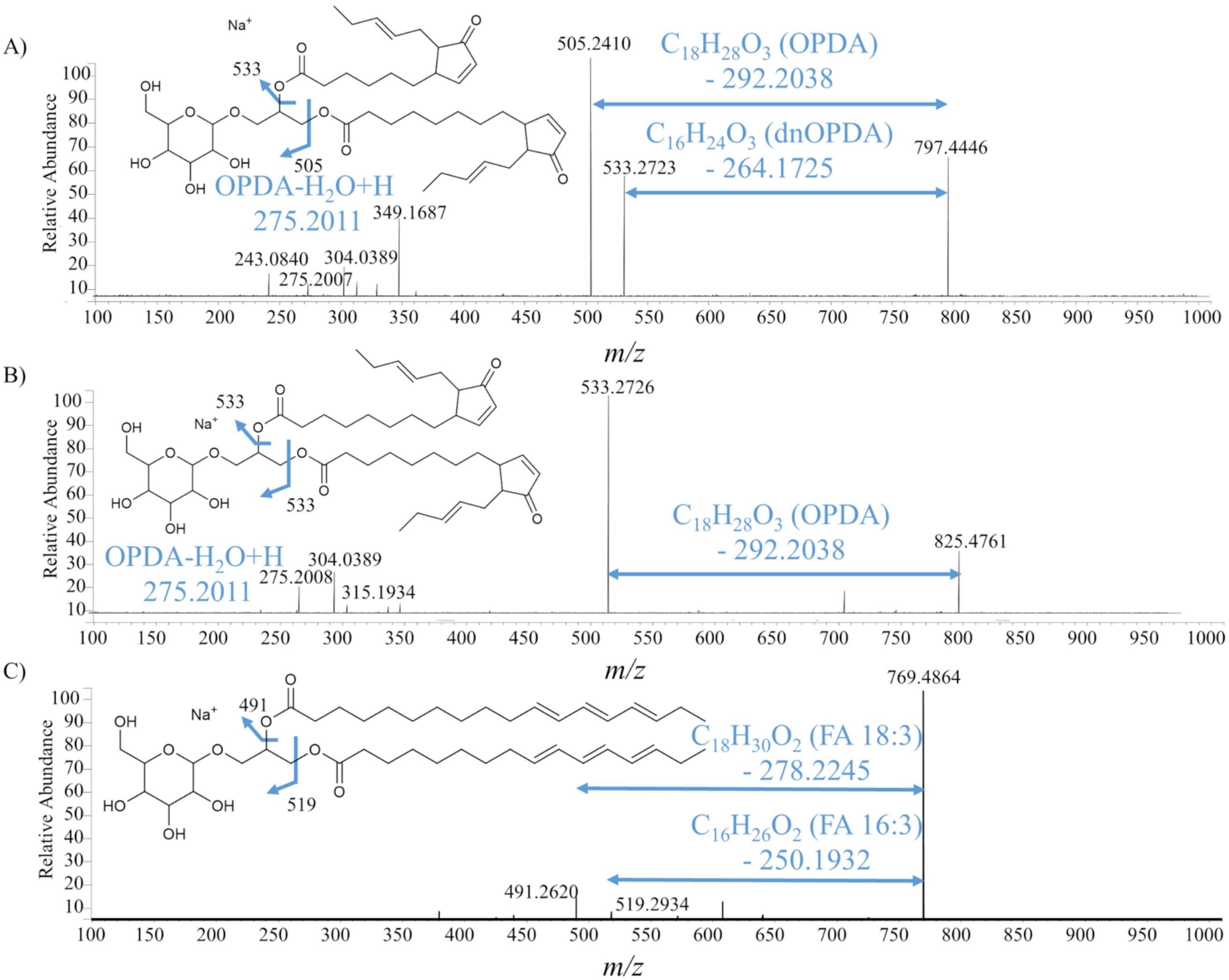

Supplementary Figure S1. MS/MS spectra of (A) arabidopside A, (B) arabidopside B, and (C) MGDG 34:6 in positive ion mode. All species are detected as Na^+ adducts.

A)

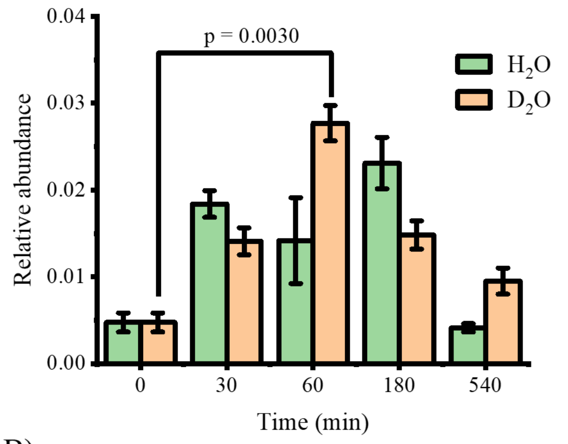

B)

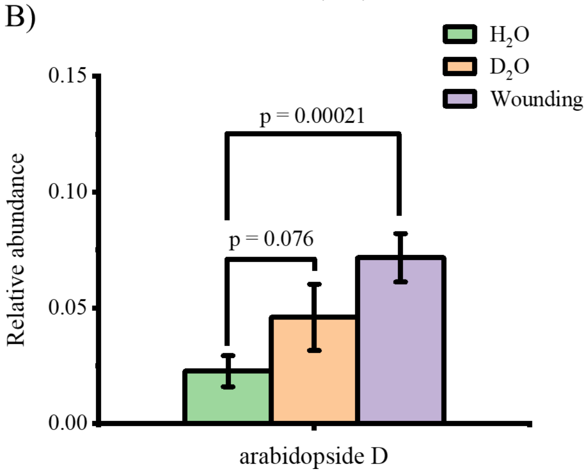

Supplementary Figure S2. Change in relative abundances of (A) arabidopside D in *A. thaliana* after moving to H_2O or 35% D_2O medium (n = 3). (B) Comparison of relative abundances of arabidopside D one hour after moving to new media vs 15 min wounding (n = 7). Arabidopside D abundance was normalized by its precursor, DGDG 36:6. Arabidopside D and DGDG 36:6 were all detected as Na^+ adduct.

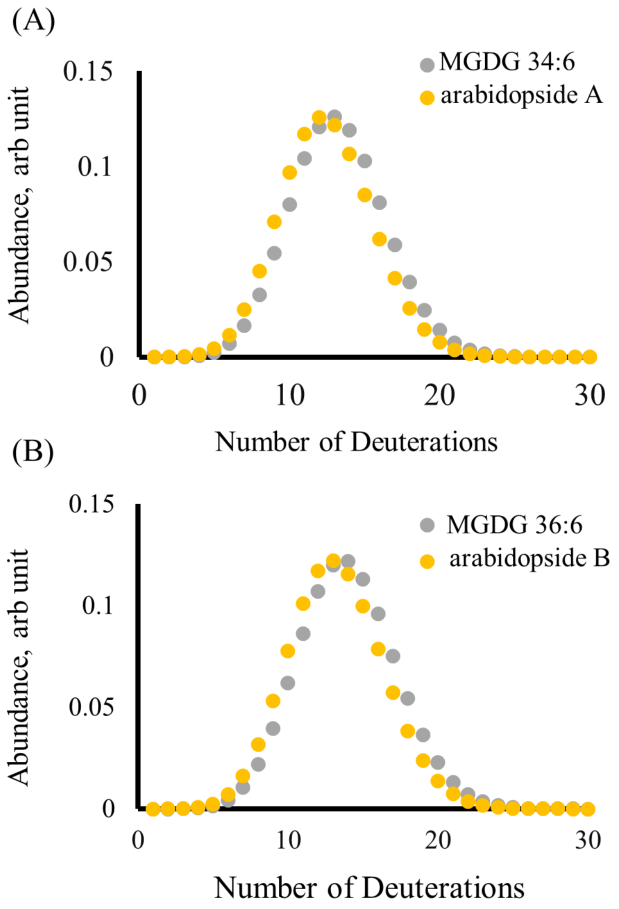

Supplementary Figure S3. D-labeling isotopologue distribution simulation for (A) MGDG 34:6 vs arabidopside A and (B) MGDG 36:6 vs arabidopside B. Binomial distribution was simulated assuming p of 0.1855 (35% D_2O concentration x 53% D-labeling efficiency) and n (the number of carbon-bound hydrogens) of 66, 62, 70, and 66 for MGDG 34:6, arabidopside A, MGDG 36:6, and arabidopside B, respectively.

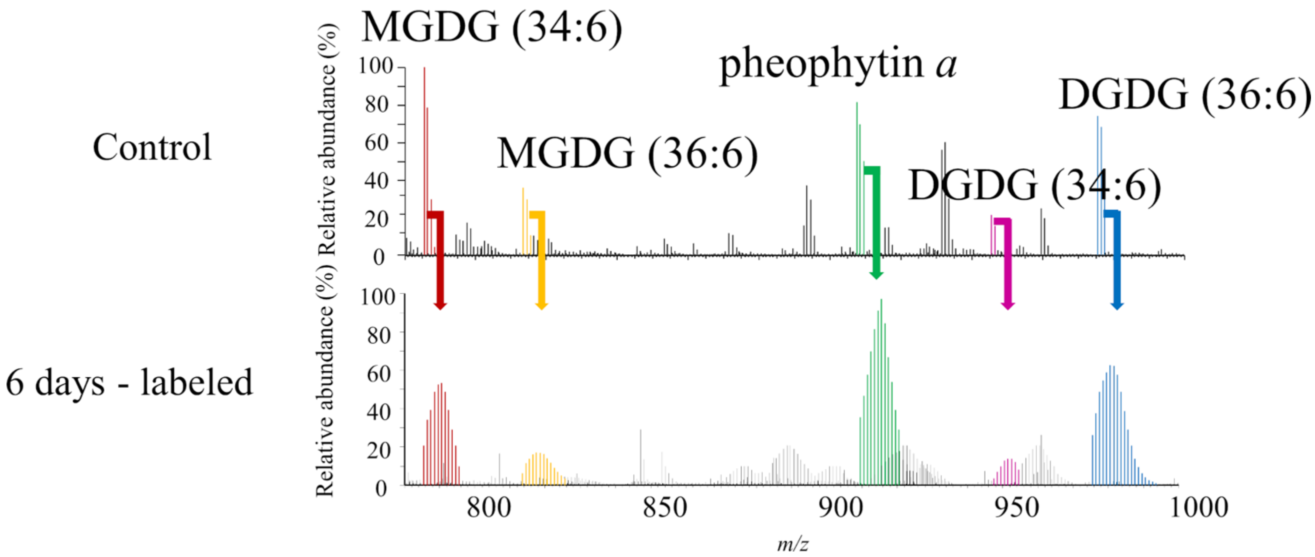

Supplementary Figure S4. Mass spectra of chloroplast lipids on *A. thaliana* leaf, showing the shift of mass spectral features due to the deuterium incorporation after growing in 35% D_2O medium for 6 days.

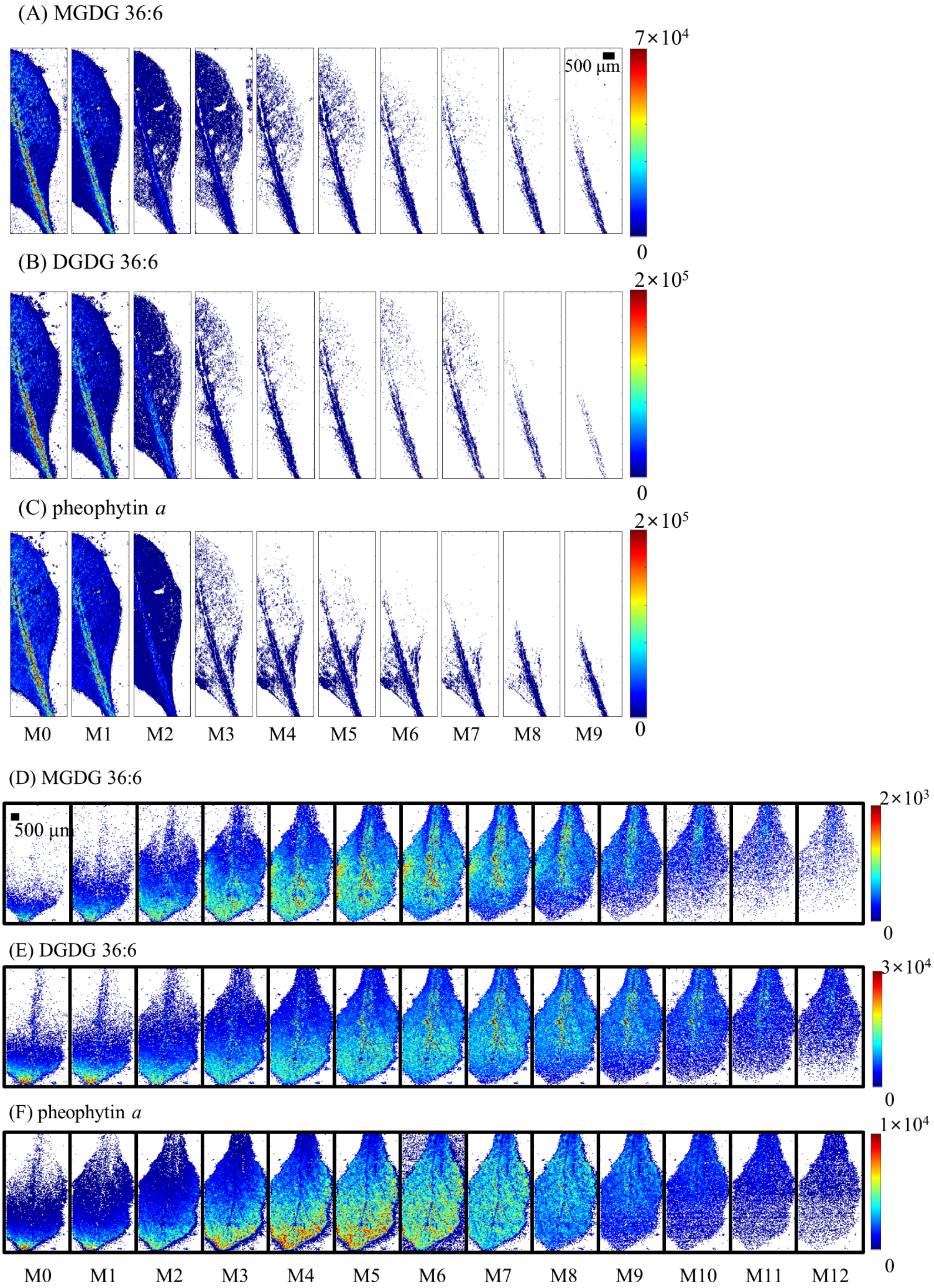
(A) MGDG 36:6
7×10^4
0
500 μm
(B) DGDG 36:6
2×10^5
0
(C) pheophytin a
2×10^5
0
M0
M1
M2
M3
M4
M5
M6
M7
M8
M9
(D) MGDG 36:6
500 μm
2×10^3
0
(E) DGDG 36:6
3×10^4
0
(F) pheophytin a
1×10^4
0
M0
M1
M2
M3
M4
M5
M6
M7
M8
M9
M10
M11
M12

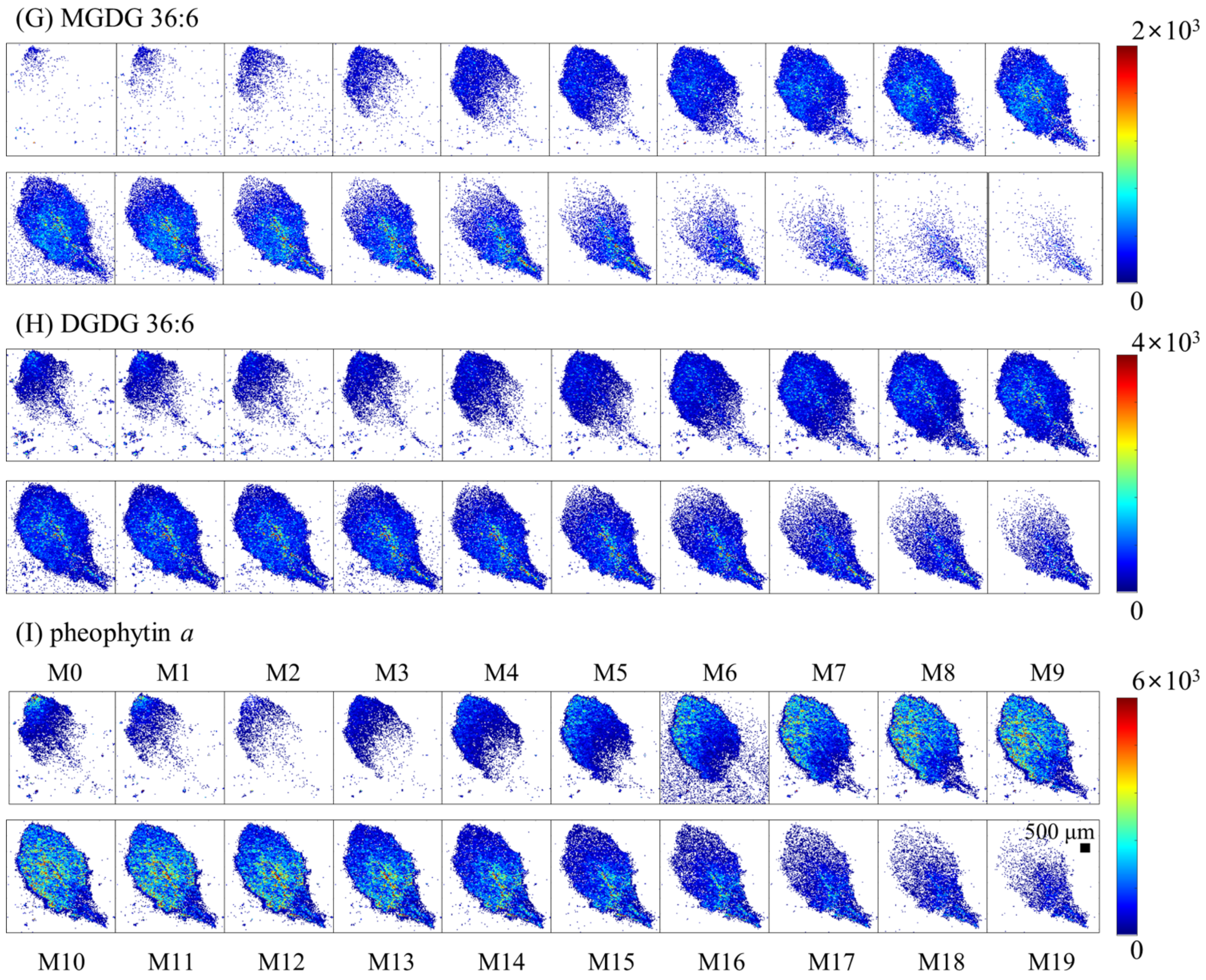

Supplementary Figure S5. MS images of (A, D, G) MGDG 36:6, (B, E, H) DGDG 36:6, and (C, F, I) pheophytin *a* in a fractured fourth leaf of *A. thaliana* grown in 35% D_2O for (A-C) 3 days, (D-F) 6 days or (G-I) 12 days on DAS 28. All detected as K^+ adduct.

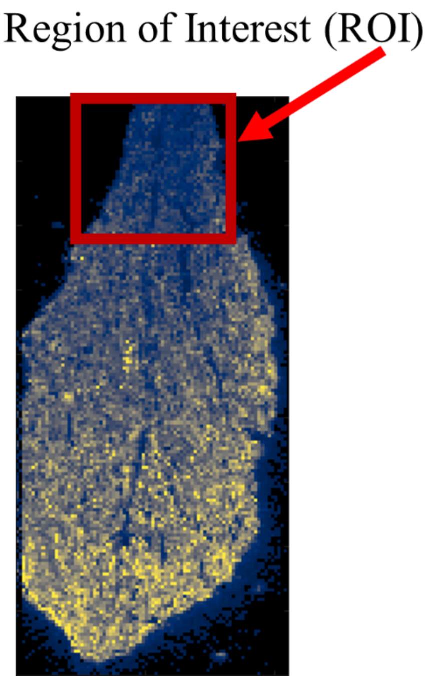

Supplementary Figure S6. Selection of region of interest (ROI). Average MS spectra were acquired for the ROI and used to calculate the D-labeling efficiency.

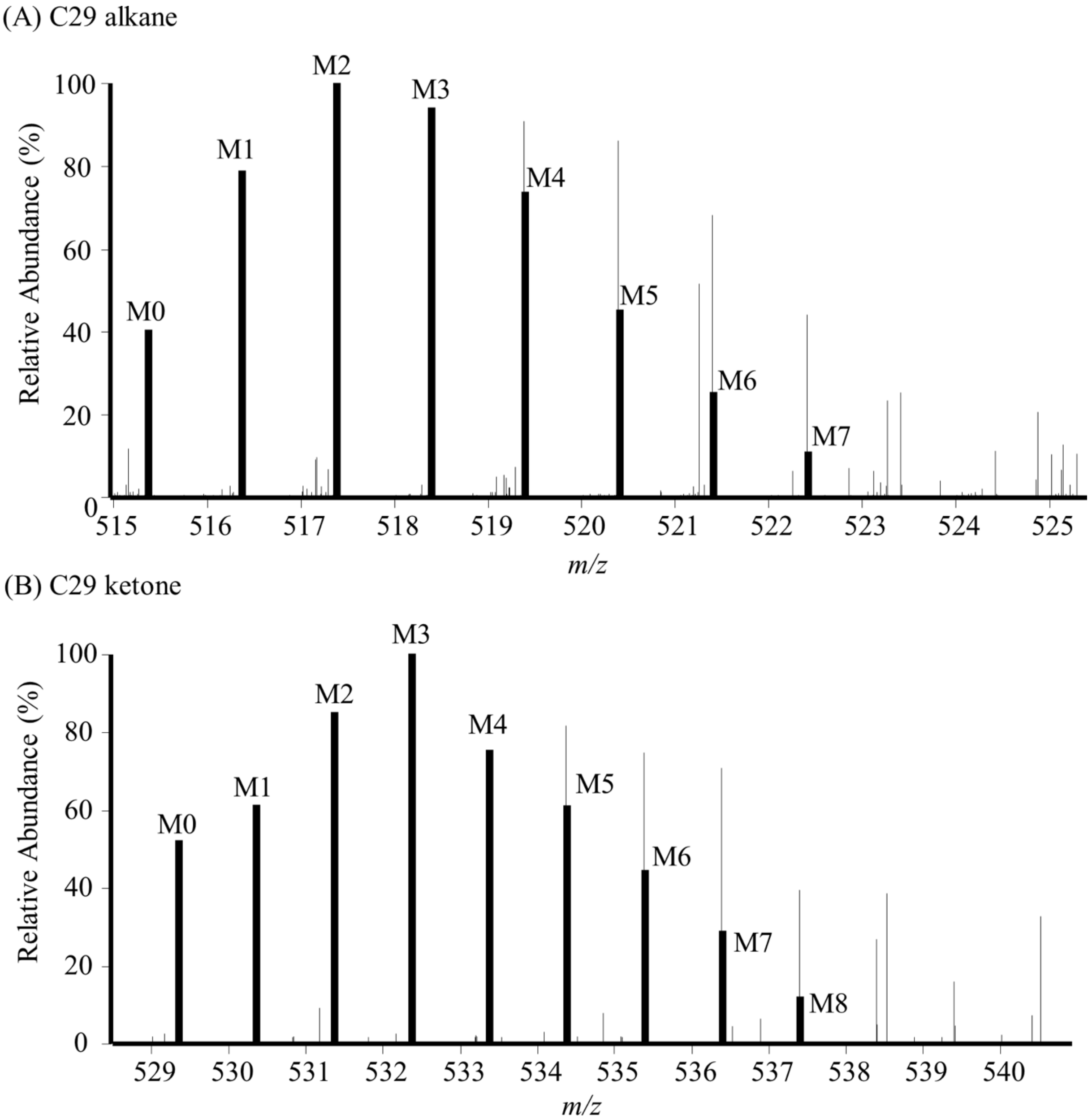

Supplementary Figure S7. Mass spectra showing a series of D-labeled (A) C29 alkane and (B) C29 ketone. $^{107}Ag^{+}$ adduct peaks are shown as bold.

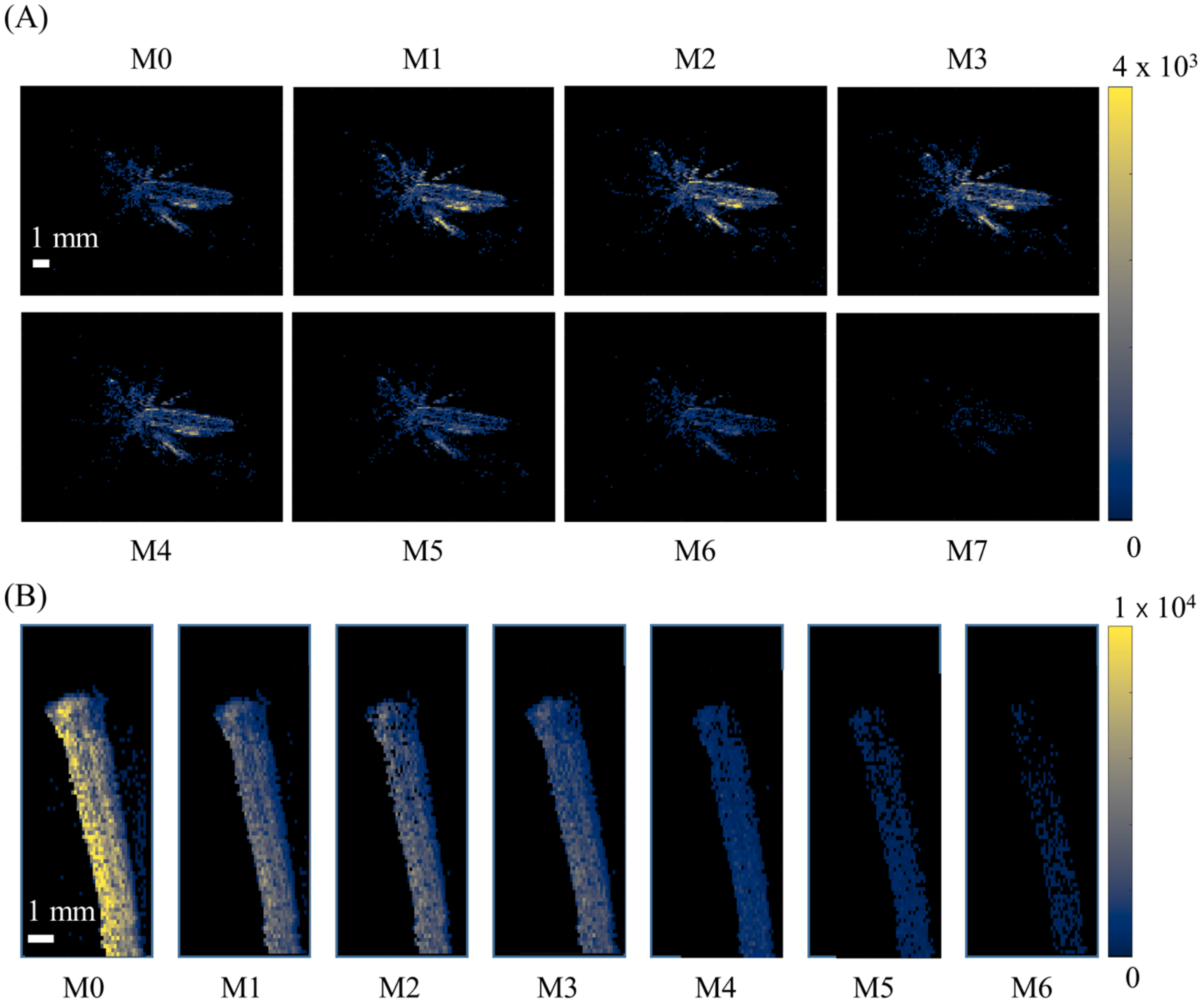

Supplementary Figure S8. MS images of deuterated [C29 ketone+^{107}Ag]$^{+}$ with various deuterations on (A) the carpel region of the flower and (B) middle section of the stem.
